# From Research to Clinical Settings: Validation of the Affect in Play Scale – Preschool Brief Version in a Sample of Preschool and School Aged Italian Children

**DOI:** 10.3389/fpsyg.2017.00728

**Published:** 2017-05-12

**Authors:** Daniela Di Riso, Silvia Salcuni, Adriana Lis, Elisa Delvecchio

**Affiliations:** ^1^Dipartimento di Psicologia dello Sviluppo e della Socializzazione, Università degli Studi di PadovaPadova, Italy; ^2^Dipartimento di Filosofia, Scienze Sociali, Umane e della Formazione, Università di PerugiaPerugia, Italy

**Keywords:** Affect in Play Scale-Preschool (APS-P), brief version, children play, tool validation, factor structure

## Abstract

Affect in Play Scale-Preschool (APS-P) is one of the few standardized tools to measure pretend play. APS-P is an effective measure of symbolic play, able to detect both cognitive and affective dimensions which classically designated play in children, but often are evaluated separately and are scarcely integrated. The scale uses 5 min standardized play task with a set of toys. Recently the scale was extended from 6 to 10 years old and validated in Italy preschool and school-aged children. Some of the main limitations of this measure are that it requires videotaping, verbatim transcripts, and an extensive scoring training, which could compromise its clinical utility. For these reasons, a Brief version of the measure was developed by the original authors. This paper will focus on an APS-P Brief Version and its Extended Version through ages (6–10 years), which consists “*in vivo*” coding. This study aimed to evaluate construct and external validity of this APS-P Brief Version and its Extended Version in a sample of 538 Italian children aged 4-to-10 years. Confirmatory factor analysis yielded a two correlated factor structure including an affective and a cognitive factor. APS-P-BR and its Extended Version factor scores strongly related to APS-P Extended Version factor scores. Significant relationships were found with a divergent thinking task. Results suggest that the APS-P-BR and its Extended Version is an encouraging brief measure assessing pretend play using toys. It would easily substitute the APS-P and its Extended Version in clinical and research settings, reducing time and difficulties in scoring procedures and maintaining the same strengths.

## Introduction

Research and theory on play have recognized that it is a significant activity that promotes children’s socio-emotional and cognitive development, enhancing also their psychological adjustment (Morrison, 2001). According to their age, children engage first in functional and exploratory play behaviors, then they shift to symbolic or pretend play (Lillard, 2001; Cartwright, 2004). Pretend play considered children’s exploration and interpretation of the world with symbols, fantasy, make-believes, expression of emotions and capacity to explore different circumstances in an imaginary context. In pretend play, children have also the chance to act out personal inner and social situations facilitating emotional and cognitive understanding of their experiences (Fein, 1987; Moore and Russ, 2006; McAloney and Stagnitti, 2009). As Marcelo and Yates (2014) pointed out, pretend play is a powerful mechanism that includes the interplay of various dimensions, all crucial for a healthy development in childhood. The use of imagination and affects expression is strictly connected with children’s gains in cognitive and representational abilities (Bornstein et al., 2002), self-regulation (Berk et al., 2006), coping (Goldstein and Russ, 2000), psychological adjustment, problem solving (Singer and Singer, 1990) and perspective taking (Fisher, 1992; Seja and Russ, 1999). Pretend play is the result of cognitive and affective integration which is responsible for children’s development and acquisition of all the abilities reported above (Russ, 2004, 2006).

In order to observe and evaluate these numerous dimensions related to children pretend play, research in this field underscored the need for standardized measures to assess children’s cognitive and affective processes that occur together in play sessions (e.g., Russ, 1993, 2004; Kaugars and Russ, 2009). However, studies often devised tools devoted to separately assess cognitive and affective domains in play; moreover, most of these instruments did not define clear standard task or administration procedures (e.g., Gitlin-Weiner et al., 2000; Chessa et al., 2012). To date, Russ (2004) was one of the first authors who tried to satisfy these conditions in devising the Affect in Play Scale (APS), a standardized instrument of pretend play in school-age children (6 to 10-years-old). It consists of a videotaped participants’ 5-min play task with two puppets and some colored blocks, scored on cognitive (Organization, Elaboration, Imagination, Comfort) as well as on affective components (the Frequency and Variety of affective themes and Positive versus Negative affective themes) of pretend play. This measure showed good psychometric properties as internal consistency, reliability and validity (Seja and Russ, 1999; Russ, 2004 for a review). A confirmatory factor analysis (CFA) in an Italian sample showed the existence of two correlated factors: a cognitive and an affective one (Delvecchio et al., 2016a). More recently, Kaugars and Russ (2009) validated an adapted form of the APS for children from 4 to 5 years, the Affect in Play Scale-Preschool version (APS-P). Differences between APS and APS-P concerns play material- stuffed toys versus puppets- and instructions, in order to motivate and engage younger children in the play session. Moreover, there are slightly differences also in the coding procedure to better adapt the coding with preschool children way of play (Delvecchio et al., 2016b). Also for APS-P, interrater reliability, internal consistency, as well as internal and external validity resulted robust (Kaugars and Russ, 2009; Fehr and Russ, 2014; Marcelo and Yates, 2014). Fehr and Russ (2014) reported also an exploratory factor analysis on APS-P that included two correlated factors. Delvecchio et al. (2016b) evaluated APS-P external and construct validity in Italian preschoolers. A two-factor model CFA approach led to two correlated factors: a cognitive and an affective one.

Although no theoretical issues might lead to the hypothesis that toys fit better for preschoolers than for school-age children, and vice-versa, the two version of the scale maintained initially two separated pathways.

Later, APS and APS-P were preliminary compared in the Italian context with the simultaneous administration of the two versions of the scale group of children aged 4-to-10 years (Mazzeschi et al., 2008). No significant differences between the two version of the play task across ages were found, excepting for Comfort: both preschool and school-age children seemed more comfortable with toys than with puppets. Authors suggested that maybe in the Italian culture children are not so familiar with puppets and, moreover, that plastic and stuffed toys might elicit a wider range of emotional expressions (Mazzeschi et al., 2008). Following these preliminary ideas, the extension of APS-P, defined as APS-P Extended Version was firstly validated in a large sample of 6-to-10 years old children and later a study using APS-P and APS-P Extended Version was run to assess pretend to play in preschool and school Italian children aged 4–10 (Delvecchio et al., 2016a,c). Findings of the two studies confirmed the good psychometric properties of this extension and the adequacy of the original APS-P structural model across the ages. The opportunity to have the same set for play session across the ages seems to give a good chance to monitor developmental changes in children assessment and psychotherapy (Cordiano et al., 2008). Moreover, in real life, school-age children use to play with toys similar to the ones proposed in the APS-P task.

Assessment of cognitive and affective abilities in pretend play in preschool and school years should be seen as beneficial for researchers and clinicians (Bergen, 2002; Pellegrini, 2010). The use of APS and APS-P, in its different versions, allowed to include tools devised on an evidence-based approach in the assessment field, since that they are standardized and validated measures of pretend play (Kazdin, 2005; Mash and Hunsley, 2005). Recent attention has been directed toward the development of Brief versions of the APS [Affect in Play Scale–Brief Rating (APS–BR); Cordiano et al., 2008] and the APS-P [Affect in Play Scale–Preschool–Brief Rating (APS-P-BR); Pearson et al., 2008; Fehr and Russ, 2014]. These measures would support and encourage to use play assessment in a wider variety of research and clinical areas because they are less invasive for patients, not requiring videotaping but *in vivo* coding and are easier to use for experts since they do not involve extensive scoring training (Cordiano et al., 2008).

The APS-BR and APS-P-BR and its Extended Version continue to involve the observation of a 5-min standardized play task, with puppets or toys to be used respectively with school and preschool children. Instructions and task prompts are the same of the original measures, except that the instructions for the brief versions do not mention videotaping. Some studies explored psychometric properties, in terms of reliability and validity, of APS-BR (Cordiano et al., 2008) and APS-P-BR and its Extended Version (Fehr and Russ, 2014), in two small samples, comparing the relationship between standard and brief version using both videotaped and *in vivo* coding. The authors of both studies concluded that the APS-BR and the APS-P-BR and its Extended Version strong correlations with the original version and with external criterion demonstrated that they are worthy measures and might be used in clinical settings in combination or in substitution to the original APS and APS-P. However, the use of two different versions for preschool and school children does not allow the continuity of measure of play across age.

The first aim of this paper was to fill this gap by validating the brief version of the APS-P and its Extended Version in an Italian sample aged 4–10 years. The second aim of this paper was to compare APS-P and its Extended Version, and APS-P-BR and its Extended Version to verify if the brief form would easily substitute the original one while maintaining the integrity of the original scale. Finally, another aim was to contribute to the external validity of the APS-P-BR and its Extended Version by relating the scale with a measure of divergent thinking.

As a preliminary result, interrater reliability was expected to be excellent, since it always resulted so in previous studies on different versions of APS-P in Italy (Delvecchio et al., 2016a,c) and APS-P-BR (Fehr and Russ, 2014). In Fehr and Russ (2014) study on the APS-P-BR and its Extended Version, just an exploratory factor analysis (EFA) was carried out yielding a two correlated factors structure: the first one included organization, imagination, and positive affect and the second one was characterized by undefined affect, comfort, and negative affect. For this reason, the first purpose was to assess the structural validity of APS-P-BR and APS-P-BR Extended Version through CFA, comparing two different models, the classical APS theoretical model (Model 1; Cordiano et al., 2008) and the one proposed by Fehr and Russ (2014) (Model 2). In Model 1, the four cognitive scores are included in the cognitive factor; frequency and positive/negative tone of affective themes load on the affective one; in Model 2 the first factor include organization, imagination, and positive affect and the second one is characterized by undefined affect, comfort, and negative affect. Furthermore, the structural invariance across age groups was investigated by mean of the multiple confirmatory factor analysis (MCFA) approach. Gender as well as age effects were explored. According to previous studies on the different versions of the APS-P, no significant gender differences were expected, however, significant differences were expected between preschool and school age children (Kaugars and Russ, 2009; Delvecchio et al., 2016a,c). It was expected that school-age children score higher both in cognitive and affective variables, demonstrating a higher ability to elaborate and organize their stories and to express a wider range and frequency of affects (e.g., Denham et al., 1994; Stagnitti et al., 2007). The last purpose was to evaluate APS-P-BR and APS-P-BR Extended Version external validity with APS-P and its Extended Version and a measure of divergent thinking, as already used in the original studies. Consistent correlations between corresponding factor scores on the APS-P and its Extended version and APS–P–BR and its Extended Version were expected (Cordiano et al., 2008; Fehr and Russ, 2014). Correlations with divergent thinking were expected significant for both the cognitive and affective factors, although with a medium to low effect size (Russ and Schafer, 2006; Cordiano et al., 2008; Delvecchio et al., 2016a,c).

## Materials and Methods

### Participants

The participants were a community sample of 538 Italian children (261 boys, 277 girls) aged 4–10 years (*M* = 6.61, *SD* = 2.20), recruited in 10 kindergartens and 8 elementary schools in urban and suburban districts in Northern regions of Italy. Participants were homogeneously distributed for gender and age [χ^2^(6) = 7.98, *p* = 0.23]. Specifically, 239 children were preschool kindergarten children aged 4–5 years old (44.42%) and 299 children were elementary school children aged 6–10 years old (55.58%). All the children included in the were Caucasian and none of them was repeating the grade. Twelve subjects were immigrants, and their data were not included in the dataset, because of their different ethnic background. Family socioeconomic status was measured using the SES scores by Hollingshead (1975). Eighty percent of the families were middle-class (i.e., SES level 3), 16% came from a high socioeconomic context (i.e., SES levels 4 and 5) and 4% were from economically disadvantaged families (i.e., SES levels 1 and 2).

### Procedure

In this study tapes of children play recruited in Delvecchio et al. (2016c) paper were employed, using the APS–P- BR and its Extended Version coding system. A total of 10 raters were employed, all of them already trained in the APS-P and its Extended Version scoring systems. They were then trained on the Brief version scoring according to the original manual (Russ, 2014) and practiced scoring on the same subsample of 40 tapes; then, the raters met with an expert in APS-P BR and its Extended Version coding system in order to discuss any general questions about scoring. Finally, raters were asked to score the scale on play sessions in tapes as they were “*in vivo*” settings, possibly without seeing more than one time each play session. They were allowed to rewind the tape just in the case they did not hear what the child on the tape was saying, simulating a request made to a child to repeat something not heard in an *in vivo* observation. Each rater used the rewind option on 10% of the tapes scored. The research procedure followed the Italian Psychologist Association (AIP) and Helsinki declaration ethical guidelines.

### Measures

#### Affect in Play Scale-Preschool and Affect in Play Scale-Preschool Extended Version

The Affect in Play Scale-Preschool (APS-P; Russ, 2004; Kaugars and Russ, 2009), and its Extended Version (Delvecchio et al., 2016a), are semi-structured 5-min videotaped play tasks, that evaluated assessing affective themes and cognitive dimensions (affect, imagination, organization, and comfort) in children 4-to-10 years old. It is based on empirically validated administration procedure and scoring attribution (Russ, 2004). Children are asked to play with a set of stuffed and plastic toys representing animals (dog, elephant, bear, shark, bunny, camel, cheetah, hippopotamus, and giraffe), and objects (a plastic car, three plastic cups, and a “hairy” rubber ball). The variety of toys is intended to elicit a wide range of emotional expressions such, for example, sadness or aggression. Each child is introduced by the following instructions: “*That’s all the toys in the basket. Now we’re going to make up a story using the toys on the table. You can play with the toys anyway that you like and have them do something together, like the bear looking for some food or play house or go to the store. Be sure to talk out loud so I can hear you. The video camera will be on so that I can remember what you say and do. You will have five minutes to play with the toys. I’ll tell you when to stop. Now, remember to play with the toys and make up a story*” (Russ, 2004, p. 19). For preschool children, the first part of the instructions are slightly modified to motivate and facilitate the task, and standardized prompts are given. Children are also informed when they have 1 min left to play. Six primary scores are scored using a detailed scoring manual (Russ, 1993, 2004; Delvecchio et al., 2016a) for the APS-P and its Extended Version: Organization, Elaboration, Imagination and Comfort, Variety and Frequency of Affective Themes. The first four scores theoretically refer to the cognitive dimension of the task construction, while the other refer to the affective domain and the emotional expression of play. Cognitive scores are coded on a five-point Likert scale. Organization assesses the complexity of the story, quality of the plot, and coherence of the narrative. Scores vary from a scenario in which disjointed and unrelated events are proposed to the well-integrated plot. Elaboration assesses variety and complexity of embellishment in the stories, use facial expressions or sound effects. Scores varied from very basic stories with no details and with play sessions with embellishment across many dimensions. Imagination assesses fantasy and number of transformations, which makes the story novel and unique. Scores vary from level of absence of symbolism to the presence of many transformations and fantasy. Comfort measures the child’s ability to be engaged in the play task, his involvement, and enjoyment in the play session. Scores vary from a reticent attitude during the play and a very comfortable child. In the APS-P and APS-P Extended version, as suggested also by Fehr and Russ (2013), affect scores were assessed through a frequency count. The frequency of Affect Expression counts affects expressed by the child in the play narrative and classifies the content according to 12 different categories including an undefined category added to assess affect expression that does not match with the other ones. Affect is scored both in the case it is expressed in the play (e.g., “Elephant takes care of the bunny”) or when affect-laden content is referenced (e.g., “This is a hammer”). Affect scores can be summed to form Total affect, Negative affect (aggression, anxiety/fear, sadness/hurt, frustration/disappointment/dislike, oral aggression, anal) and Positive affect (nurturance/affection, happiness/pleasure, competition, oral, sexual), and scores. The Variety of Affect Score is the number of different affect categories expressed by the child during the play. It can also be summed to form Total affect variety, Positive affect variety, and Negative affect variety scores. Interrater reliability reported excellent values, as well as internal consistency, reported values from satisfactory to excellent for both the versions of the scale (Cordiano et al., 2008; Kaugars and Russ, 2009; Chessa et al., 2011; Fehr and Russ, 2013). CFA of APS-P and APS-P Extended Version showed adequate fit for the two factors solution original model designed by Russ (2004).

#### The Affect in Play Scale- Preschool Brief Version and its Extended version (Russ, 2004; Fehr and Russ, 2014)

A manual with description of the instructions, prompts, and scores is available (Russ, 2004). To facilitate decision for scoring, brief descriptions of each anchor point are given in the manual. The purpose of the manual was not to overload the rate with many examples that would be difficult to filter through during the observation but to provide a context for scoring the play observation.

APS-P-BR and APS-P-BR Extended Version instructions and their rating scales are based on the ones of the APS-P and its Extended version: while observing the child tapes, the rater scores both the cognitive and affective aspects of his/her play with alterations made for ease of scoring in a live brief rating observation. Five scores were obtained: Organization, Imagination, Comfort, Frequency of Affect, and positive and negative Tone. All dimensions were scored on a Likert scale, ranging from 1 to 4 to avoid “hiding” in the middle (Fishman and Galguera, 2003; Streiner and Norman, 2003), and brief descriptions of each anchor point are given in the manual. A major difference between the original APS-P and its Extended Version and the new APS–P-BR and its Extended Version was the way in which the Frequency of Affect Expression is scored. In the APS-P and its Extended Version, the *Frequency of Affect Expression* score is designed to measure the amount of affect expression, defined in affective units and displayed within the play session. In the APS-P-BR and its Extended Version, instead of a total frequency count, the rater is asked to rate the total frequency of affect on a scale ranging from 1 (*low*) to 4 (*high*) affect expression. The APS–P- BR did not produce a score for a variety of affect categories because the focus was on rating the relative positive/negative tone of the affect expression and not on the specific affect categories. The 11 affect categories are defined just to familiarize the rater with what constitutes a unit of affect expression. Scores can range from *Low* (1; 0–2 affect units) to *High* (4; >15 affect units). In addition, the APS–P-BR and its Extended Version ask the observer to rate the “overall tone of affect in the story, based on the average amount of positive or negative affect expression in the affect units in the child’s play.” The Tone score on the APS–P-BR and its Extended Version corresponded with the positive and negative affect scores on the original APS-P and its Extended Version. The Tone variable measures the proportion of positive to negative affect expressed in the play and measures the overall affective tone of the story. The tone is based on the estimated amount of positive and negative affect units. The rater is again instructed to keep an estimated tally of the positive and negative affect units. Scores range from predominately negative affect dominating the play (1) to predominately positive affect dominating the play (4). The numeric changes made for the APS–P-BR and its Extended Version do not result in a significant deviation from the conceptual format of the original APS-P and its Extended Version, with the total amount of affect expressed, the tone of the affect expressed, and the quality of the fantasy scored in the play sample.

#### Alternate Uses Task

Assesses divergent thinking based upon Wallach and Kogan’s (1965) adaptation of Guilford’s Alternate Uses Task. Children were asked to think of as many alternatives as possible for six everyday life objects (newspaper, button, box, car tire, key, shoe, knife). Two separate scores were calculated (1) Fluency, the number of acceptable uses generated by the child and (2) Flexibility, the number of different categories of use generated by the child. The Alternate Uses Task shows good reliability and validity in many studies conducted with children (Kogan, 1983; Runco, 1991).

### Data Analysis

At first, the interrater reliability among 10 raters was assessed on 40 protocols. Interrater reliability was determined using ICC with a 95% confidence interval. A CFA (Maximum Likelihood method) was run to evaluate the structural validity of the APS-P-BR and its Extended Version. Following Van de Schoot et al. (2012) suggestions, those fit indices were considered for model evaluation: Root Mean Square Error of Approximation (RMSEA), Comparative Fit Index (CFI), and Tucker–Lewis Index (TLI). The Bayesian Information Criterion (BIC) index was used to compare alternative models In order to test APS-P-BR and its Extended Version measurement invariance across age (preschool versus school-aged children) a MCFA was run. At first, the hypothetical model was assessed on preschool and school-age children separately, after that configural invariance, metric invariance, and scalar invariance were performed. In order to test for evidence of invariance (1) overall model fit, (2) BIC, and (3) change in CFI (ΔCFI) between constrained models were examined. Invariance is supported by the presence of adequate overall fit, lower BIC, and ΔCFI ≤ 0.01 between increasingly constrained models (Vandenberg and Lance, 2000; Cheung and Rensvold, 2002; Chen, 2007; Thienot et al., 2014).

After the establishment of the scalar invariance, MANOVA were performed on the APS-P BR and its Extended Version factor mean scores with gender and age as between-subject variables. Pearson’s correlations were carried out between factor scores of play tasks and divergent thinking to assess APS-P-BR and its Extended Version external validity. R Development Core Team (2012), Package lavaan (Rosseel, 2012) and the PASW Statistics 18 (SPSS Inc., 2009) were used.

## Results

Inter-rater reliability was assessed on 40 randomly selected videos of APS-P and its Extended Version scored with the APS-P-BR and its Extended Version scoring system. Ten independent raters (Ph.D. level and graduate students in clinical psychology), one of which trained by the author of the scale, rated the 40 protocols independently. The average ICCs showed adequate to excellent range: organization: 0.82–0.88, elaboration: 0.86–0.90, imagination: 0.81–0.86, comfort: 0.88–0.94, frequency of affects: 0.84–0.90, and positive/negative tone of affect expression: 0.86–0.89. The remaining protocols were coded independently by the raters (about 50 protocols each).

Means and standard deviations for the total sample, preschool children (4 and 5 years old), school-aged children (from 6 to 10 years old) as well as boys and girls are reported in **Table 1**.

**Table 1:** Means and standard deviations for all the variables of the APS-P Brief and APS-P Brief Extended Version for the total sample, gender, and age (*N* = 538).

			Gender				Age			
				
	Overall sample (*N* = 392)		Boys (*n* = 261)		Girls (*n* = 277)		4–5 years (*n* = 239)		6–10 years (*n* = 299)	
					
	*M*	*SD*	*M*	*SD*	*M*	*SD*	*M*	*SD*	*M*	*SD*
Organization	2.34	0.94	2.28	0.98	2.40	0.91	1.86	0.80	2.73	0.87
Elaboration	2.29	1.00	2.23	1.01	2.36	0.98	2.08	1.02	2.46	0.94
Imagination	2.37	0.88	2.39	0.92	2.36	0.84	2.07	0.88	2.62	0.79
Comfort	3.17	0.79	3.10	0.81	3.24	0.78	3.05	0.87	3.27	0.71
Frequency of affects	3.64	0.77	3.64	0.77	3.65	0.77	3.40	0.96	3.84	0.50
Tone of affect expression	2.92	0.88	2.82	0.89	3.03	0.86	2.83	1.11	3.00	0.63


Fit indices comparing the classical APS-P theoretical model (Model 1; Cordiano et al., 2008) and the model proposed by Fehr and Russ (2014) (Model 2) are displayed in **Table 2**. Model 1 included the four cognitive scores loading in the cognitive factor and frequency and positive/negative tone of affective themes loading in the affective one; whereas Model 2 included organization, imagination, and positive affect in the first factor and undefined affect, comfort, and negative affect in the second.

**Table 2:** Goodness of fit indices of APS-P Brief and APS-P Brief Extended Version for Model 1 and Model 2.

Goodness of fit indexes categories	Fit indexes	Model 1 (theoretical model)	Model 2 (two factors; Fehr and Russ, 2014)	Good fit	Acceptable fit
*Df*		8	8		
Satorra–Bentler scaled chi-square		40.31	92.74	0 ≤ χ^2^ ≤ 2*df*	2*df* < χ^2^ ≤ 3*df*
Descriptive measures of overall model fit	RMSEA	0.078	0.14	0 ≤ RMSEA ≤ 0.05	0.05 < RMSEA ≤ 0.08
Descriptive measures	TLI	0.958	0.81	0.95 ≤ TLI ≤ 1.00	0.90 ≤ TLI < 0.95
based on model comparison	CFI	0.977	0.90	0.95 ≤ CFI ≤ 1.00	0.90 ≤ CFI < 0.95
Descriptive measures of model parsimony	Model BIC	10245.24	14930.32	Smaller than BIC for comparison model	


*RMSEA, Root Mean Square Error of Approximation; TLI, Tucker–Lewis Index; CFI, Comparative Fit Index; BIC, Bayesian Information Criterion.*

**FIGURE 1:**
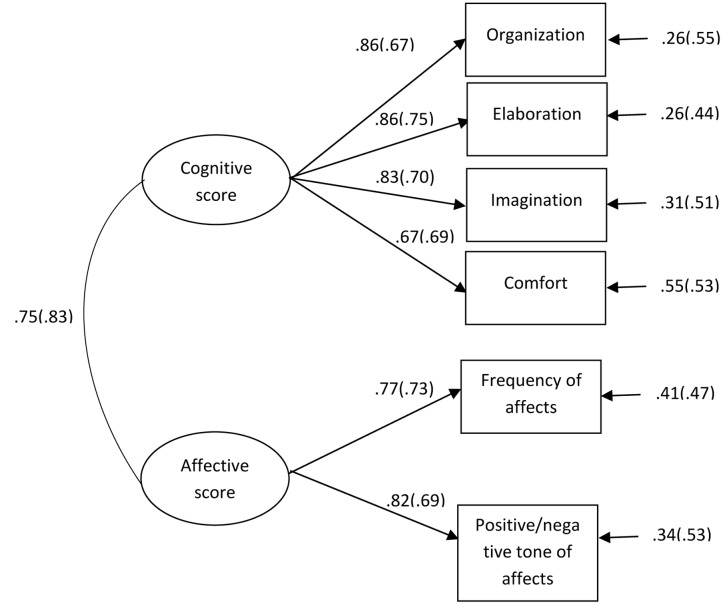
**Dimensional structure of APS-P Brief and APS-P Brief Extended Version, Model 1.** APS-P Brief Extended version values are in brackets.

The parsimonious index was lower for Model 1 suggesting that the theory-driven model fits the data more adequately than the Model 2. **Figure 1** displayed the factor loadings of Model 1; they were all significant and comprised between 0.67 and 0.80. The inter-factor correlation between the cognitive and affective factors was 0.82.

**Table 3** reports the fit indexes for both preschool and school-aged children as well as the results of the MCFA. Fit indexes referring to the subsample of school-age children showed an excellent fit, as well as for preschool children with the exception of RMSEA that showed slightly higher value than expected. Configural model (M1) indices displayed adequate fit. Looking at the metric invariance (M2), ΔCFI was equal to 0.01 and BIC was lower in comparison to M1 (**Table 3**). Testing for scalar invariance (M3), ΔCFI showed greater value than the suggested cut off and BIC resulted higher than for M2 (**Table 3**). Moreover, M3 showed a poor fit. These findings advocated for a weak measurement invariance of the hypothesized model across preschool and school-aged children. Thus, following Van de Schoot et al. (2012) recommendation, it is suggested to proceed testing for partial invariance by freeing one item at the time. As proposed by Dimitrov’s (2010), it is acceptable freeing less than 20% of parameters, which correspond to less than 2 variables for APS-P-BR and its Extended Version. Examination of the modification indices highlighted item 1 (“organization”) as the most problematic, suggesting to free its intercepts across groups in order to reach a better fit for the model. Therefore, the partial scalar invariance model (M3a) was run. It showed generally an adequate fit. Results displayed evidence of partial invariance between the groups showing values of ΔCFI below the cut off and a lower BIC value (**Table 3**) between M3a and M2.

**Table 3:** Test of measurement invariance of the APS-P Brief and APS-P Brief Extended Version across age (*N* = 538).

	χ^2^	*df*	*p*	RMSEA	TLI	CFI	ΔCFI	BIC
Baseline model: preschoolers (*n* = 239)	25.23	8	0.001	0.095	0.958	0.978		
Baseline model: school-aged (*n* = 299)	21.08	8	0.007	0.074	0.957	0.977		
M1: configural invariance	46.30	16	<0.001	0.084	0.957	0.977		10066.76
M2: metric invariance	64.66	20	<0.001	0.091	0.950	0.968	0.009	10059.55
M3: scalar invariance	152.35	24	<0.001	0.141	0.880	0.904	0.064	10122.29
M3a: partial scalar invariance	84.41	23	<0.001	0.092	0.946	0.958	0.010	10048.44


*RMSEA, Root Mean Square Error of Approximation; TLI, Tucker–Lewis Index; CFI, Comparative Fit Index; BIC, Bayesian Information Criterion.*

The analysis of variance was run to assess age and gender differences. Mean levels of the APS-P-BR and APS-P-BR Extended Version factor scores, for the total sample, preschool and school-aged children, as well as boys and girls, are displayed in **Table 4**.

**Table 4:** Means and standard deviations for the APS-P Brief and APS-P Brief Extended Version Cognitive and Affective factors for the total sample, gender, and age.

			Gender				Age			
				
	Overall sample (*N* = 538)		Boys (*n* = 261)		Girls (*n* = 277)		Preschool (*n* = 239)		School-age (*n* = 299)	
					
	*M*	*SD*	*M*	*SD*	*M*	*SD*	*M*	*SD*	*M*	*SD*
Cognitive factor	0.01	0.68	-0.04	0.69	0.04	0.67	-0.26	0.69	0.21	0.59
Affective factor	0.01	19.61	0.31	18.68	-0.29	20.49	-2.64	20.85	2.11	18.33


Results showed that school-aged children scored higher than preschoolers in the cognitive factor (*F*_(1,534)_ = 71.29, *p* < 0.001, $\eta_{p}^{2}$ = 0.118) as well as in the affective factor (*F*_(1,534)_ = 8.06, *p* < 0.001, $\eta_{p}^{2}$ = 0.015). No gender differences [cognitive factor: (*F*_(1,534)_ = 1.83, *p* = 0.176, $\eta_{p}^{2}$ = 0.003); affective factor: (*F*_(1,534)_ = 0.08, *p* = 0.774, $\eta_{p}^{2}$ = 0.000)] as well as no interaction effects [cognitive factor: (*F*_(1,534)_ = 0.06, *p* = 0.800, $\eta_{p}^{2}$ = 0.000); affective factor: (*F*_(1,534)_ = 0.93, *p* = 0.336, $\eta_{p}^{2}$ = 0.002)] between gender and age were found.

Correlations between APS-P-BR and APS-P-BR Extended Version factor scores and the factor scores of the divergent thinking measure were all significant (**Table 5**). However, different patterns emerged. APS-P-BR and its Extended Version cognitive factor showed higher positive correlations with both the cognitive and affective factors of APS-P and its Extended Version, whereas the affective one displayed a medium correlation with the cognitive APS-P and its Extended Version factor and a lower value for the affective one. In line with what expected, fluency and flexibility (divergent thinking) showed significant positive correlations with both cognitive and affective domains of play.

**Table 5:** Correlations of the APS-P-BR and its Extended Version with APS-P and its Extended Version and divergent thinking.

		APS-P and its Extended Version		Divergent Thinking	
			
		Cognitive factor	Affective factor	Fluency	Flexibility
APS-P-Br and its Extended Version	Cognitive Factor	0.949^∗∗^	0.383^∗∗^	0.231^∗∗^	0.288^∗∗^
	Affective Factor	0.848^∗∗^	0.095^∗^	0.109^∗^	0.235^∗∗^


*^∗∗^*p* < 0.001; ^∗^*p* < 0.05.*

## Discussion

This paper evaluated the construct and external validity of this APS-P-BR and its Extended Version through age, in a wide sample of preschool and school age Italian children. The coding system of the brief version of the APS-P was applied to the videotapes of children play sessions recruited in the Delvecchio et al. (2016a) study, in which APS-P and its Extended Version standard procedure was administered. CFAs results supported the best data fit for the theoretical two-correlated-factor model with one factor related to cognitive dimension and one factor to affective dimension (Cordiano et al., 2008; Kaugars and Russ, 2009). Previous papers focusing on the original form of the APS-P and its Extended Version in Italian children confirmed empirically the same structure, both for preschooler and school-age children (e.g., Delvecchio et al., 2016a,c). On the same direction, MCFA showed some evidence of measurement consistency across preschool and school-aged children; metric invariance was partial and obtained after freeing organization variable. Although most of the fit indices confirmed this adequacy, some others did not, advocating for further studies on structural validity and metric invariance of APS-P-BR and its Extended Version. Moreover, though Fehr and Russ (2014), run an EFA on APS-P-BR in USA, as far as we know, this was the first attempt to conduct a CFA on the APS-P-BR and its Extended Version. As for age, significant differences were found for both cognitive and affective factors. As expected, also for the APS-P-BR and its Extended Version school-age children reported higher scores then preschooler (Kaugars and Russ, 2009; Delvecchio et al., 2016a,c). This finding is in line with previous literature on play, which underlined how older children showed a more sophisticated way of play, in terms of their abilities to structure and organize their stories and to comprehend and express a wider range of affective themes (e.g., Stagnitti et al., 2007). According to gender, no significant differences were found for the cognitive and the affective factors. These findings were in line with expectations and with Kaugars and Russ’ (2009) and Delvecchio et al. (2016a) studies in which no gender differences emerged.

As expected, significant correlations between cognitive and affective factors of the brief version of the APS-P and its Extended Version and the correspondent factors of the original version were found (Cordiano et al., 2008; Fehr and Russ, 2014). However, while observing correlations patterns in detail, Cohen’s d was high for the one between the two cognitive factors, instead it was low for the correlation between the affective factor of the brief version and of the original one. This data suggests that the coding systems of APS-P-BR and its Extended Version and APS-P and its Extended Version for cognitive variables are substantially able to detect the same aspect of play, maybe because the scoring of the four cognitive variables consists of a global score. In this case, the possibility to rewind the tape in the APS-P and its Extended Version does not add information about the cognitive scores that can be easily detected also in the *in vivo* coding. On the other hand, the affective variables could be affected by the possibility to watch the tape more than one time. The affective variables score is based on counting each word, expression or object in the story conveying affective themes, and during the *in vivo* coding some of them can be lost. Moreover, it is interesting to note that the APS-P-BR and its Extended Version cognitive factor correlates with high Cohen’s d with both cognitive and affective factors of the APS-P original version and its Extended Version, showing also a correlation between cognitive factor of the brief version and affective factors of both brief and original version of APS-P and their Extended Version. On the other hand, APS-P-BR and its Extended Version affective factor showed medium correlations with both cognitive and affective factors of the APS-P original version and its Extended Version, confirming that the affective factor has a quite different trend in the brief version, for the reasons hypothesized before. Significant positive correlations between APS-P-BR and its Extended Version cognitive and affective factors and divergent thinking were found. Children with higher scores in cognitive and affective components of play reported higher score in both Fluency and Flexibility. The same pattern was found in studies involving the APS-P original version and its Extended Version, suggesting that both cognitive and affective dimension of pretend play are associated with improved divergent thinking skills (e.g., Russ and Schafer, 2006; Marcelo and Yates, 2014; Delvecchio et al., 2016a).

This paper contributes to the validation of APS-P-BR and its Extended Version, a measure that would encourage the use play assessment in a wider variety of research and clinical areas, not requiring videotaping. This aspect makes the measure less invasive for patients, also increasing their confidentiality. Results suggest that APS-P-BR and its Extended Version through age, is a reliable and valid measure in terms of construct and external validity, retracing the main results obtained also in the APS-P and its Extended Version (e.g., Kaugars and Russ, 2009; Delvecchio et al., 2016a,c).

The APS-P-BR and its Extended Version do not completely overlap the original form, especially for the affective dimension. As discussed before, the affective factor of the brief version did not exactly retrace the one of the original version. So, on the one hand, the APS-P-BR and its Extended Version could be easily used in clinical settings, not requiring videotaping, on the other hand, APS-P and its Extended Version could be more suitable to have a complete affective profile of the play session. For sure the APS-P-BR and its Extended Version is a promising tool for assessing children pretend play that would easily substitute or, in some cases, used together with the APS-P and its Extended Version through age.

The present study showed also some limitations. First, APS-P-BR is not administered directly to children, but its scoring system was applied to tapes of children play sessions following APS-P and its Extended Version standard procedure. Future studies need to be carried out using APS-P-BR and its Extended Version procedure and without videotaping. Then, raters who scored the tapes with the brief version procedure were already familiar with the APS-P and its Extended Version coding system. This aspect could have affected their way of scoring in terms of make the two coding processes more similar. Then, although the study involved a large sample of children from 4 to 10 years old, generalizability is limited to non-clinical sample and to Italian culture, stating that culture could affect children way of play (Chessa et al., 2012). Future research should involve different kinds of clinical and cross-cultural samples. Finally, APS-P- BR and its Extended Version affective factor would need specific attention. Training should deepen the scoring system of affective variables of the brief version because they seem to require a moderate amount of time to be practiced. Expertise in play session scoring resulted strictly connected with an increasing availability of the raters and accuracy in detecting cognitive, but primarily, affective variables. Future development of the APS-P-BR and its Extended Version scoring procedure could include an affective theme checklist the raters could use during the *in vivo* administration to be guided in detecting the highest numbers of affective themes.

## Ethics Statement

Comitato Etico Area 17, Università degli Studi di Padova. As explained in the manuscript, in these studies videotapes of already recruited children play sessions were coded. Following, I reported the consent procedure used for the original recruitment. This study was conducted in compliance with the ethical standards for research outlined in the Ethical Principles of Psychologists and Code of Conduct (American Psychological Association, 2010). Participation in the study was solicited via leaflets. School approval and parents written signed informed consent to participate in the study were obtained before data collection. Children were asked to provide their own oral consent. No incentives were awarded and voluntary participation was emphasized. Administration was proposed during scheduled classes, according to the standard administration procedures. Confidentiality was assured by replacing children’s personal information with a numeric code. According to their teachers, all children were developing typically. Before starting the task, preschoolers’ cognitive and verbal abilities were assured by the WPPSIIII (Wechsler, 2008) vocabulary and bloc design subtests. A score equal or greater than was required in order to participate in the study. At the same time, for school -aged children, cognitive and verbal abilities were confirmed by teachers’ reports on the basis of Italian language and maths tests.

## Author Contributions

DDR supervised the rater who coded the videotapes. She wrote the theoretical introduction and the discussion of the results. SS supervised the objective/hypothesis and procedure sessions. AL supervised the sample recruitment and the discussion of the results. ED supervised the sample recruitment and carried out statistical analysis.

## Conflict of Interest Statement

The authors declare that the research was conducted in the absence of any commercial or financial relationships that could be construed as a potential conflict of interest.
